# Sickness absence and associations with sociodemographic factors, health risk behaviours, occupational stressors and adverse mental health in 40,343 UK police employees

**DOI:** 10.1017/S2045796024000283

**Published:** 2024-05-07

**Authors:** S. Parkes, P. Irizar, N. Greenberg, S. Wessely, N. T. Fear, M. Hotopf, S. A. M. Stevelink

**Affiliations:** 1 King’s Centre for Military Health Research, Institute of Psychiatry, Psychology & Neuroscience, King’s College London, London, UK; 2 Department of Sociology, School of Social Sciences, University of Manchester, Manchester, UK; 3 Academic Department of Military Mental Health, Institute of Psychiatry, Psychology & Neuroscience, King’s College London, London, UK; 4 Department of Psychological Medicine, Institute of Psychiatry, Psychology & Neuroscience, King’s College London, London, UK; 5 South London and Maudsley NHS Foundation Trust, London, UK

**Keywords:** Airwave Health Monitoring Study, mental health, police, sick leave, sickness absence

## Abstract

**Aims:**

Police employees may experience high levels of stress due to the challenging nature of their work which can then lead to sickness absence. To date, there has been limited research on sickness absence in the police. This exploratory analysis investigated sickness absence in UK police employees.

**Methods:**

Secondary data analyses were conducted using data from the Airwave Health Monitoring Study (2006–2015). Past year sickness absence was self-reported and categorised as none, low (1–5 days), moderate (6–19 days) and long-term sickness absence (LTSA, 20 or more days). Descriptive statistics and multinomial logistic regressions were used to examine sickness absence and exploratory associations with sociodemographic factors, occupational stressors, health risk behaviours, and mental health outcomes, controlling for rank, gender and age.

**Results:**

From a sample of 40,343 police staff and police officers, forty-six per cent had no sickness absence within the previous year, 33% had a low amount, 13% a moderate amount and 8% were on LTSA. The groups that were more likely to take sick leave were women, non-uniformed police staff, divorced or separated, smokers and those with three or more general practitioner consultations in the past year, poorer mental health, low job satisfaction and high job strain.

**Conclusions:**

The study highlights the groups of police employees who may be more likely to take sick leave and is unique in its use of a large cohort of police employees. The findings emphasise the importance of considering possible modifiable factors that may contribute to sickness absence in UK police forces.

## Introduction

In 2022, there were approximately 225,000 full-time equivalent (FTE) police employees in England and Wales and 164,000 police officers in the United Kingdom (Allen and Mansfield, 2022). Between 2010 and 2019 there was a 19% real-terms reduction in police funding from government and local services (National Audit Office, 2018). This resulted in lower police officer numbers (Allen and Mansfield, 2022) and likely contributed to increased occupational stress for an already overburdened workforce (Burchell *et al.*, 2022). Policing is often considered a stressful occupation due to high rates of exposure to traumatic situations, such as responding to homicides and road traffic accidents (Cartwright and Roach, 2020; Violanti *et al.*, 2016). However, traumatic events are often not reported as the most frequent causes of work-related stress by police staff (Elliott-Davies, 2021b). Instead, the most commonly cited sources of stress are a high workload, poor work–life balance, working shifts, being uncertain about a future role or career (Elliott-Davies, 2021b), a lack of support, long working hours, job demand (sustained physical/mental effort) and job pressure (Purba and Demou, 2019). These stressors have demonstrated a strong relationship with adverse mental health outcomes, including psychological distress, and emotional exhaustion (Purba and Demou, 2019). Furthermore, research has shown that occupational stress is a strong risk factor for depression, suicidal ideation and post-traumatic stress disorder (PTSD) in police employees (Syed *et al.*, 2020).

Exposure to stressors, traumatic or otherwise, can be associated with police officers taking sick leave. In 2021, 1,965 FTE police officers were on long-term sick leave (more than 28 days) across 43 police forces in England and Wales which accounted for 1.5% of the uniformed workforce (Home Office, 2021). In 2020, 48% of police officers in the UK reported 1 or more days of sickness absence and 32% of those attributed at least 1 day to stress, depression or anxiety (Elliott-Davies, 2021a). Similarly, in 20 UK police forces over the last 10 years, 56% of police officers took sick leave due to stress and 37% due to anxiety and depression (Cartwright and Roach, 2020). Further, 39% of police officers who had one period of sickness absence because of mental ill-health or work-related stress took additional absences from work (Cartwright and Roach, 2020). Occupational stress for police officers has also been associated with increased consumption of alcohol (Houdmont and Jachens, 2021), which may lead to sickness absences (Marzan *et al.*, 2022).

Previous research reported police employees were on long-term sick leave due to musculoskeletal and stress-related issues (Her Majesty’s Inspectorate of Constabulary (HMIC), 1997). However, a recent report on the UK general population highlighted mental health conditions as one of the top four reasons for taking sick leave (Office for National Statistics, 2022). To date, there has been limited research conducted on sickness absence in police employees and its relation to occupational stressors, health risk behaviours and mental health. The existing research has often focused on specific areas such as shift work (Fekedulegn *et al.*, 2013), harassment, threats or violence (Svedberg and Alexanderson, 2012) or gender differences (Körlin *et al.*, 2009).

This exploratory analysis aimed to investigate the associations between sickness absence in UK police employees with sociodemographic factors, occupational stressors, health risk behaviours and mental health outcomes.

## Method

### Study design and sample

Cross-sectional data were obtained from the Airwave Health Monitoring Study (AHMS), an occupational cohort study, which aimed to determine the possible health risks associated with Terrestrial Trunked Radio (TETRA) usage in the UK police forces (Elliott *et al.*, 2014). TETRA is a digital communication system used by the police forces and other emergency services in the UK since 2001 (Elliott *et al.*, 2014). Participating police force employees completed an enrolment questionnaire during routine administration or when they visited occupational health services, and a health screen. The enrolment questionnaire included questions on sociodemographic characteristics, work environment, lifestyle and health (including mental health and sickness absence). The health screen was conducted by trained nurses and included an interview which collected data on medical history, smoking status and alcohol consumption. Data were collected between April 2006 and March 2015 and this analysis is based on a sample of 40,343 police employees (Elliott *et al.*, 2014).

### Measures

#### Sickness absence

The main outcome of interest was the amount of self-reported sickness absence days taken within the year before screening. Days of sickness absence were grouped into four categories based on the spread of the data and similar to a previous study using the AHMS data (Irizar *et al.*, 2021a): *none, low* (1–5), *moderate* (6–19) and *long-term sickness absence* (LTSA, 20 or more). LTSA of 20 days or more was chosen in accordance with the UK government guidance on long-term sickness of 4 weeks or more (https://www.gov.uk/taking-sick-leave).

#### Sociodemographic variables

Sociodemographic variables included gender (male/female), rank (police officer/police staff), age, ethnicity (white/all other ethnic groups combined), marital status (married or cohabiting/divorced or separated/single/other) and educational attainment. Age was grouped into 10-year age bands from *under 30 years* to *50 years and above*, similar to previous studies that used the AHMS data (Irizar *et al.*, 2021a, 2021b; Stevelink *et al.*, 2020). Education was grouped by the AHMS into categories of *low* (O levels/General Certificate of Secondary Education (GCSEs)or none) and *high* (A levels, degree or higher).

#### Occupational variables

Occupational variables included salary, years in the police force, total number of hours worked per week (including overtime), shift work, job strain and job satisfaction. *Years in the police force* was determined by subtracting the year of joining the police from the year that the AHMS screening took place. Job strain was measured using six items from the Job Content Questionnaire (Karasek, 1985). Job satisfaction was a single-item measure: ‘Taking all things into consideration how satisfied are you with your job as a whole?’ with choices of *very satisfied, satisfied, dissatisfied* and *very dissatisfied*.

#### Health risk behaviours

Health risk behaviours included alcohol consumption (including binge drinking), smoking status (non-smoker/current smoker) and general practitioner (GP) consultations (number of times GP was consulted for health problems in the past year). Alcohol consumption was determined by the AHMS using a drinks diary, which recorded the drinks consumed in the past week for beer, spirits, white wine, red wine and fortified wine (converted into units). Binge drinking was defined as having six or more drinks on one occasion and frequent binge drinking was binge drinking at least two to four times a month, the same as a previous study (Irizar *et al.*, 2021a). GP consultations were categorised based on the spread of the data (0, 1–2, 3–4, ≥5). Participants were asked whether they were smoking at the time of screening, so *non-smoker* may include those who previously smoked.

#### Mental health

Mental health measures included those for probable depression, anxiety and PTSD. Probable depression was measured using the nine-item Patient Health Questionnaire (PHQ-9) (Kroenke *et al.*, 2001) with response options from *not at all* (0) to *nearly every day* (3) and a score of 10 or above indicated probable depression (Spitzer *et al.*, 2006). Probable anxiety was measured using the anxiety subscale of the Hospital Anxiety and Depression Scale (HADS-A) and a score of 11 or above indicated probable anxiety (Zigmond and Snaith, 1983). Probable PTSD was measured using the Trauma Screening Questionnaire (TSQ) with response options from *not at all* to *extremely* (Brewin *et al.*, 2002). Any response other than *not at all* was scored as one and a score of six or above indicated probable PTSD (Brewin *et al.*, 2002). The TSQ was only asked to participants who answered yes to the following question: ‘Have you been exposed to trauma in the past 6 months?’ (*n* = 5,460). Any participant who answered no to this question was assigned to the non-caseness group for PTSD.

All three mental health measures have been widely used and shown to have good reliability and validity (Bjelland *et al.*, 2002; Brewin, 2005; Brewin *et al.*, 2002; Kroenke *et al.*, 2001; Zigmond and Snaith, 1983). The internal reliability of the measures in the current sample was good, PHQ-9 *α* = 0.84, HADS-A *α* = 0.83, TSQ *α* = 0.92.

### Data analysis

Data were analysed using Stata 17 (StataCorp., College Station, TX, USA). Descriptive statistics were reported for the sample characteristics using frequencies and percentages to describe the distribution of sociodemographic factors, health risk behaviours, occupational stressors and mental health outcomes across the whole sample and by sickness absence status. To compare variables, chi-squared tests for categorical variables and ANOVA for the comparison of continuous variables means were used. Multinomial logistic regression was used to explore any potential associations with sickness absence and sociodemographic factors, health risk behaviours, occupational stressors and mental health outcomes. These were adjusted for known confounders from the literature including age, gender and rank (Cartwright and Roach, 2020; Fekedulegn *et al.*, 2013). To account for multiple testing, the data were corrected using the false discovery rate (Benjamini and Hochberg, 1995) on all variables and the results were the same with and without this correction (data not shown). Unadjusted odds ratio (Table S1, supplementary materials) and adjusted odds ratio (AOR) are reported with 95% confidence intervals (CIs). Statistical significance is reported using *p* values with a threshold of *p* < 0.05.

### Missing data

There was a high amount of missing data for the shift work variables *shift working* (73%, *n* = 29,319) and *control over shift patterns/≤9 hours of rest between shifts* (84%, *n* = 33,837) (Table 1). This was due to the shift work questions only being included in later versions of the AHMS assessment. Therefore, the shift work variables were only used to describe the sample. The other variables with the highest amount of missing data were *rank* (10%, *n* = 3,855), *GP consultations* (7%, *n* = 2,799) and *probable PTSD* (6%, *n* = 2,508). Given that the data were assumed to be not missing at random and the amount of missingness on the variables of interest was low (<10%), it was not deemed appropriate to use multiple imputation (Sterne *et al.*, 2009). Therefore, data were excluded from specific analyses where it was missing by listwise deletion (Hughes *et al.*, 2019; Pepinsky, 2018). Data on the variables of interest were available for 41,082 respondents. A total of 739 respondents were excluded from our analyses if their rank was reported as *other* as it could not be determined if they were employed in the police force as either police staff or police officers. The final sample available for analytical purposes included 40,343 police employees.

### Ethics and data access

The AHMS received ethical approval from the National Health Service multi-site research ethics committee (MREC/13/NW/0588). Written informed consent was obtained from all subjects.

The AHMS data were securely accessed through the Dementias Platform UK (application number 0378) and data access agreements were completed for each member of the research team.

## Results

### Sample characteristics

The sample size was 40,343, including 71% police officers and 29% police staff (Table 1). The majority were male (63%), aged between 40 and 49 years (40%), identified as white (95%) and were married or cohabiting (78%). Most of the sample reported being at low risk for alcohol consumption (55%) and were binge drinkers for less than two to four times a month (70%). Only 9% of the sample were smokers. For mental health outcomes, the prevalence of probable PTSD was 4%, probable depression 10% and probable anxiety 8%. Job strain levels varied from low (27%), high (24%), active (28%) and passive (21%). Most participants worked 40 hours or less (42%) and were shift workers (59%).

**Table 1.** Sociodemographic factors, health risk behaviours, occupational stressors and mental health outcomes of police employees

Characteristic	Total (*N* = 40,343)	*n* (%)	Missing *n* (%)
Rank	36,488		3,855 (9.56)
Police officer		26,075 (71.46)	
Police staff		10,413 (28.54)	
Gender	40,339		4 (0.01)
Male		25,421 (63.02)	
Female		14,918 (36.98)	
Age (in years)	40,343		0 (0)
<30		5,280 (13.09)	
30−39		13,199 (32.72)	
40−49		15,942 (39.52)	
≥50		5,922 (14.68)	
Ethnicity	40,056		287 (0.71)
White		37,958 (94.76)	
All other ethnic groups combined		2,098 (5.24)	
Marital status	40,084		259 (0.64)
Married/cohabiting		31,155 (77.72)	
Divorced/separated		3,240 (8.08)	
Single		4,767 (11.89)	
Other		922 (2.30)	
Education	40,084		259 (0.64)
Low (O levels/GCSEs or none)		13,389 (33.40)	
High (A levels, degree or higher)		26,695 (66.60)	
Salary	40,084		259 (0.64)
<£25,999		8,497 (21.20)	
£26,000–£37,999		16,658 (41.56)	
£38,000–£59,999		13,657 (34.07)	
>£60,000		1,272 (3.17)	
Total hours worked per week (inc. overtime)	39,621		722 (1.79)
≤40 hours		16,547 (41.76)	
41−48 hours		13,351 (33.70)	
≥49 hours		9,723 (24.54)	
Years in police force	40,278		65 (0.16)
≤5 years		8,245 (20.47)	
6−10 years		9,110 (22.62)	
11−20 years		12,520 (31.08)	
>20 years		10,403 (25.83)	
Shift working	11,024		29,319 (72.67)
Is a shift worker		6,506 (59.02)	
Is not a shift worker		3,528 (32.00)	
Is a shift worker but only works shifts two or three times a year		990 (8.98)	
Control over shift patterns	6,506		33,837 (83.87)
Flexibility to decide shifts to work		324 (4.98)	
Work to pre-planned duty roster		5,738 (88.20)	
Other		444 (6.82)	
≤9 hours of rest between shifts	6,506		33,837 (83.87)
Almost never		990 (15.22)	
Seldom		922 (14.17)	
Sometimes		1,608 (24.72)	
Often		1,885 (28.97)	
Always		1,101 (16.92)	
Smoking status	40,074		269 (0.67)
Non-smoker		36,568 (91.25)	
Current smoker		3,506 (8.75)	
Alcohol consumption	40,060		283 (0.71)
Non-drinker		3,659 (9.13)	
Low risk		22,120 (55.22)	
Hazardous		13,070 (32.63)	
Harmful		1,211 (3.02)	
Binge drinking	40,061		282 (0.70)
Binge drinks less than two to four times a month		27,933 (69.73)	
Binge drinks at least two to four times a month		12,128 (30.27)	
GP consultations (in the past year)	37,544		2,799 (6.94)
0		11,436 (30.46)	
1−2		18,209 (48.50)	
3−4		5,263 (14.02)	
≥5		2,636 (7.02)	
Probable PTSD (TSQ ≥ 6)	37,835		2,508 (6.22)
Yes		1,477 (3.90)	
No		36,358 (96.10)	
Probable depression (PHQ-9 ≥ 10)	39,621		722 (1.79)
Yes		3,875 (9.78)	
No		35,746 (90.22)	
Probable anxiety (HADS-A ≥ 11)	39,621		722 (1.79)
Yes		3,351 (8.46)	
No		36,270 (91.54)	
Job satisfaction	39,621		722 (1.79)
Very satisfied		8,155 (20.58)	
Satisfied		24,219 (61.13)	
Dissatisfied		5,922 (14.95)	
Very dissatisfied		1,325 (3.34)	
Job strain	39,621		722 (1.79)
Low		10,765 (27.17)	
High		9,595 (24.22)	
Active		11,048 (27.88)	
Passive		8,213 (20.73)	

TSQ, Trauma Screening Questionnaire; PHQ-9, Patient Health Questionnaire; HADS-A, Hospital Anxiety and Depression Scale (anxiety subscale).

### Sickness absence and sociodemographic factors

The majority of police employees in our sample reported no sickness absence in the past year (46%), followed by a low amount (33%) (Table 2). Moderate amounts of sickness absence (13%) and LTSA (8%) were less frequently reported. Women had more LTSA (10%) compared with men (7%). Police staff had more sickness absences of any amount (61%) compared with police officers (50%). Older staff had more LTSA (30–39 years old 7%, 40–49 years old 9% and 50 or over 9%), compared with those under 30 years old (5%). Police employees separated or divorced had more LTSA (11%) compared with those married or cohabiting (8%). Unadjusted models are reported in Table S1 (supplementary materials).

**Table 2.** Multinomial logistic regression for health risk behaviours, occupational stressors, mental health outcomes and sickness absence for police employees. Row frequencies and percentages are shown, along with adjusted multinomial odds ratios (AMORs) and 95% confidence intervals (CIs). Reference group for sickness absence is no sickness absence in the past year

	Days of sickness absence in the past year (*N* = 40,208)						
Characteristic	None *n* (%)	Low (1−5) *n* (%)	AMOR (95% CI)	Moderate (6−19) *n* (%)	AMOR (95% CI)	Long-term sickness absence (20 or more) *n* (%)	AMOR (95% CI)
Overall (*n* = days of sick leave)	18,572 (46.19)	13,295 (33.07)		5,189 (12.91)		3,152 (7.84)	
Ethnicity							
White	17,504 (46.18)	12,570 (33.17)	1.00	4,850 (12.80)	1.00	2,977 (7.85)	1.00
All other ethnic groups combined	967 (46.09)	657 (31.32)	0.92 (0.83, 1.03)	313 (14.92)	1.12 (0.98, 1.29)	161 (7.67)	1.01 (0.84, 1.20)
Marital status							
Married/cohabiting	14,697 (47.19)	10,236 (32.86)	1.00	3,859 (12.39)	1.00	2,355 (7.56)	1.00
Divorced/separated	1,384 (42.72)	1,033 (31.88)	1.09 (1.00, 1.20)	481 (14.85)	1.28 (1.14, 1.44)***	342 (10.56)	1.32 (1.15, 1.51)***
Single	2,033 (42.66)	1,662 (34.87)	0.86 (0.80, 0.93)***	700 (14.69)	0.98 (0.89, 1.09)	371 (7.78)	0.97 (0.85, 1.11)
Other	403 (43.76)	312 (33.88)	0.89 (0.75, 1.05)	134 (14.55)	1.00 (0.81, 1.25)	72 (7.82)	0.98 (0.75, 1.30)
Education							
Low (O levels/GCSEs or none)	6,055 (45.23)	4,225 (31.56)	1.00	1,841 (13.75)	1.00	1,265 (9.45)	1.00
High (A levels, degree or higher)	12,462 (46.70)	9,018 (33.79)	0.90 (0.85, 0.94)***	3,333 (12.49)	0.79 (0.74, 0.85)***	1,875 (7.03)	0.69 (0.64, 0.76)***
Salary							
<£25,999	3,099 (36.48)	3,381 (39.80)	1.00	1,288 (15.16)	1.00	727 (8.56)	1.00
£26,000–£37,999	6,953 (41.75)	5,781 (34.71)	0.96 (0.89, 1.03)	2,460 (14.77)	1.11 (1.01, 1.22)*	1,459 (8.76)	1.03 (0.91, 1.16)
£38,000–£59,999	7,525 (55.11)	3,878 (28.40)	0.70 (0.64, 0.76)***	1,355 (9.92)	0.62 (0.56, 0.70)***	896 (6.56)	0.58 (0.51, 0.67)***
>£60,000	940 (73.90)	203 (15.96)	0.29 (0.25, 0.35)***	71 (5.58)	0.27 (0.21, 0.36)***	58 (4.56)	0.30 (0.22, 0.41)***
Total hours worked per week (inc. overtime)							
≤40 hours	6,798 (41.09)	5,756 (34.79)	1.00	2,348 (14.19)	1.00	1,641 (9.92)	1.00
41−48 hours	6,278 (47.03)	4,491 (33.65)	0.97 (0.91, 1.03)	1,690 (12.66)	0.91 (0.84, 0.99)*	889 (6.66)	0.68 (0.61, 0.75)***
≥49 hours	5,216 (53.65)	2,864 (29.46)	0.78 (0.73, 0.83)***	1,071 (11.02)	0.76 (0.69, 0.83)***	572 (5.88)	0.54 (0.48, 0.60)***
Years in police force							
≤5 years	3,266 (39.70)	3,324 (40.40)	1.00	1,131 (13.75)	1.00	506 (6.15)	1.00
6−10 years	3,630 (39.96)	3,328 (36.63)	1.03 (0.95, 1.11)	1,363 (15.00)	1.20 (1.09, 1.33)***	764 (8.41)	1.38 (1.21, 1.58)***
11−20 years	5,813 (46.59)	4,005 (32.10)	0.86 (0.80, 0.93)***	1,611 (12.91)	0.97 (0.87, 1.07)	1,049 (8.41)	1.17 (1.02, 1.34)*
>20 years	5,840 (56.35)	2,622 (25.30)	0.62 (0.57, 0.68)***	1,076 (10.38)	0.70 (0.62, 0.79)***	826 (7.97)	0.92 (0.79, 1.07)
Smoking status							
Non-smoker	17,177 (47.02)	11,983 (32.80)	1.00	4,572 (12.52)	1.00	2,800 (7.66)	1.00
Current smoker	1,330 (37.97)	1,241 (35.43)	1.22 (1.12, 1.33)***	591 (16.87)	1.53 (1.37, 1.70)***	341 (9.73)	1.45 (1.27, 1.66)***
Alcohol consumption							
Non-drinker	1,551 (42.41)	1,161 (31.75)	0.97 (0.89, 1.06)	540 (14.77)	1.17 (1.05, 1.31)**	405 (11.07)	1.54 (1.35, 1.75)***
Low risk	10,022 (45.36)	7,569 (34.26)	1.00	2,855 (12.92)	1.00	1,647 (7.45)	1.00
Hazardous	6,379 (48.90)	4,104 (31.46)	0.98 (0.93, 1.04)	1,615 (12.38)	1.04 (0.97, 1.13)	947 (7.26)	1.05 (0.96, 1.15)
Harmful	542 (44.83)	380 (31.43)	1.10 (0.95, 1.27)	146 (12.08)	1.09 (0.89, 1.32)	141 (11.66)	1.74 (1.42, 2.14)***
Binge drinking							
Binge drinks less than two to four times a month	12,767 (45.76)	9,314 (33.39)	1.00	3,625 (12.99)	1.00	2,192 (7.86)	1.00
Binge drinks at least two to four times a month	5,727 (47.30)	3,901 (32.22)	1.02 (0.97, 1.08)	1,531 (12.65)	1.05 (0.98, 1.13)	948 (7.83)	1.11 (1.02, 1.21)*
GP consultations (in the past year)							
0	7,490 (65.49)	3,392 (29.66)	0.54 (0.51, 0.57)***	462 (4.04)	0.19 (0.17, 0.22)***	92 (0.80)	0.10 (0.08, 0.12)***
1−2	7,798 (42.82)	6,786 (37.27)	1.00	2,623 (14.40)	1.00	1,002 (5,50)	1.00
3−4	1,487 (28.25)	1,702 (32.34)	1.28 (1.18, 1.39)***	1,214 (23.07)	2.33 (2.12, 2.56)***	860 (16.34)	4.21 (3.76, 4.72)***
≥5	467 (17.72)	588 (22.31)	1.35 (1.18, 1.55)***	566 (21.47)	3.34 (2.90, 3.84)***	1,015 (38.51)	16.10 (14.05, 18.46)***
Mental health							
PTSD case	560 (37.91)	427 (28.91)	1.11 (0.97, 1.27)	259 (17.54)	1.74 (1.49, 2.05)***	231 (15.64)	2.55 (2.15, 3.02)***
Depression case	1,291 (33.32)	1,204 (31.07)	1.27 (1.16, 1.38)***	734 (18.94)	2.07 (1.87, 2.29)***	646 (16.67)	3.28 (2.94, 3.66)***
Anxiety case	1,190 (35.51)	1,060 (31.63)	1.17 (1.07, 1.29)**	587 (17.52)	1.74 (1.56, 1.94)***	514 (15.34)	2.48 (2.20, 2.80)***
Job satisfaction							
Very satisfied	4,543 (55.72)	2,377 (29.15)	0.70 (0.66, 0.74)***	777 (9.53)	0.62 (0.57, 0.68)***	456 (5.59)	0.63 (0.56, 0.71)***
Satisfied	11,111 (45.89)	8,212 (33.91)	1.00	3,103 (12.81)	1.00	1,788 (7.38)	1.00
Dissatisfied	2,204 (37.22)	2,105 (35.55)	1.33 (1.24, 1.43)***	96 (16.26)	1.61 (1.47, 1.77)***	650 (10.98)	1.87 (1.68, 2.08)***
Very dissatisfied	434 (32.75)	417 (31.47)	1.40 (1.21, 1.62)***	266 (20.08)	2.33 (1.97, 2.76)***	208 (15.70)	3.11 (2.58, 3.75)***
Job strain							
Low	5,348 (49.69)	3,502 (32.54)	1.00	1,226 (11.39)	1.00	686 (6.37)	1.00
High	3,867 (40.30)	3,396 (35.39)	1.31 (1.23, 1.40)***	1,436 (14.97)	1.53 (1.40, 1.68)***	896 (9.34)	1.72 (1.54, 1.93)***
Active	5,787 (52.39)	3,309 (29.96)	0.88 (0.83, 0.94)***	1,222 (11.06)	0.92 (0.84, 1.01)	728 (6.59)	0.97 (0.86, 1.08)
Passive	3,290 (40.07)	2,904 (35.37)	1.26 (1.17, 1.35)***	1,225 (14.92)	1.48 (1.35, 1.63)***	792 (9.65)	1.71 (1.52, 1.93)***

Adjusted for rank, gender and age.

* *p* < 0.05, **p < 0.01, ***p < 0.001.

### Sickness absence and occupational stressors

Staff who had served between 6 and 10 years had an elevated risk for LTSA (AOR: 1.38, 95% CI: 1.21, 1.58) compared with those in the police force for 5 years or less (Table 2). Police employees who were dissatisfied or very dissatisfied with their jobs were more likely to report any sickness absence compared with those who were satisfied with their jobs; effect sizes increased for greater amounts of sickness absences (Table 2). Those who were very dissatisfied with their job had three times greater odds (AOR: 3.11, 95% CI: 2.58, 3.75) of LTSA compared with those who were satisfied with their job. Police employees with passive or high job strain had an elevated risk for any sickness absence compared to those with low job strain (Table 2).

Having a higher salary (≥£38,000) and working 49 or more hours was associated with having less sick leave compared with lower salaries (<£38,000) and fewer working hours (Table 2). Those in the police force over 20 years were less likely to have low (AOR: 0.62, 95% CI: 0.57, 0.68) and moderate (AOR: 0.70, 95% CI: 0.62, 0.79) amounts of sickness absence compared with those in the police force 5 years or less. Police employees with high educational attainment and high job satisfaction were less likely to have any amount of sickness absence compared with those who reported low educational attainment and low job satisfaction (Table 2).

### Sickness absence and health risk behaviours

Smokers and those with three or more GP consultations in the past year had an elevated risk for any amount of sickness absence compared with non-smokers and those with 1–2 days of GP consultations (Table 2). Non-drinkers (AOR: 1.54, 95% CI: 1.35, 1.75) and those with harmful alcohol consumption (AOR: 1.74, 95% CI: 1.42, 2.14) had a higher risk for LTSA compared with those who had low-risk alcohol consumption. Further, frequent binge drinking was associated with a mildly elevated risk of having more LTSA compared with those who were binge drinkers less than two to four times a month (AOR: 1.11, 95% CI: 1.02, 1.21).

### Sickness absence and mental health outcomes

Police employees with probable PTSD, probable depression and probable anxiety were more likely to have moderate sickness absence and LTSA compared with those without a mental health problem (Table 2); effect sizes increased for greater amounts of sickness absence. Those with probable PTSD (AOR: 2.55, 95% CI: 2.15, 3.02) and probable anxiety (AOR: 2.48, 95% CI: 2.20, 2.80) were twice as likely to report LTSA and those with probable depression were three times as likely (AOR: 3.28, 95% CI: 2.94, 3.66).

## Discussion

### Key findings

Most police employees reported no sickness absence in the past year, followed by a low amount (1–5 days). Moderate amounts (6–19 days) of sickness absence and LTSA (20 or more days) were less frequently reported. When compared with police employees who did not have any days of sickness absence, those taking sick leave were more likely to be women, non-uniformed police staff, divorced or separated, smokers, those with three or more GP consultations in the past year and poor mental health. The factors associated with lower odds of reporting sickness absence included having a high salary, high educational attainment, working 49 or more hours, being in the police force over 20 years, having high job satisfaction and low job strain.

### Sickness absence and mental health outcomes

Adverse mental health outcomes including probable PTSD, probable depression and probable anxiety for police employees were associated with greater amounts of sickness absence compared to those without a mental health problem. This is consistent with previous research which reported police employees taking sick leave due to mental ill-health (Cartwright and Roach, 2020; Elliott-Davies, 2021a). However, due to the cross-sectional nature of our study, the direction of the relationship is not known. Rather than mental ill-health leading to sickness absence, it is possible that those taking sick leave later developed mental health problems and employment status may be a confounder (Pearce *et al.*, 2007). It is important to consider this bias when interpreting the results of our study. Furthermore, as the data for sickness absence and mental health were collected at different time points, any potential associations should be interpreted with caution. However, previous research has shown mental health measures to be relatively stable over time (Kessler *et al.*, 2005).

In our sample, police staff were more likely to take sick leave compared with police officers. Previous research reported that police staff and constables were more likely to take sick leave for psychological or mental ill-health compared with sergeants and higher ranks (Cartwright and Roach, 2020; Elliott-Davies, 2021b). This may be because police staff are exposed to more traumatic experiences over which they have no control, such as call handlers listening to distressed callers which may cause vicarious trauma (Golding *et al.*, 2017). Furthermore, police staff may have not expected to be exposed to trauma and may have not developed the coping strategies that officers have (Cartwright and Roach, 2020). However, research supporting this has collected sickness absence data using freedom of information requests and self-reporting so the data may be incomplete and prone to bias (Cartwright and Roach, 2020; Elliott-Davies, 2021a).

### Sickness absence and sociodemographic factors

Fifty-four per cent of police employees had at least 1 day of sickness absence, which is similar to the 2020 Demand, Capacity and Welfare Survey of UK police officers (Elliott-Davies, 2021a). The survey reported that 48% of police officers (*n* = 12,471) had 1 or more days of sickness absence and 32% attributed at least 1 day to stress, depression or anxiety. However, the survey only included police officers and not police staff (Elliott-Davies, 2021a).

In our study, women were more likely to report any amount of sickness absence (63%) compared with men (49%), similar to national data sources which found that women in the general population reported more sickness absences compared with men (Office for National Statistics, 2022). This gender gap in the rates of sickness absence is consistent across several Western countries but the reasons for this are unclear (Østby *et al.*, 2018). UK national statistics reported female police officers were more likely to be on long-term sick leave (1.7%) compared with men (1.3%) (Home Office, 2021). However, there is limited previous research in this area and the studies have used several different measures for sickness absence (Körlin *et al.*, 2009), therefore more investigation is required to understand the gender differences.

### Sickness absence and health risk behaviours

Smoking, abstinence from alcohol and harmful drinking were all associated with more sickness absence. Previous research reported that police employees meeting the criteria for probable depression, anxiety and/or PTSD were more likely to engage in health risk behaviours (Irizar *et al.*, 2022). The association of abstinence from alcohol with sickness absence may be related to the ‘sick quitter’ hypothesis which suggests that abstinence may be due to attempts to prevent further decline of a physical or mental health issue related to heavy drinking (Marzan *et al.*, 2022; Shaper *et al.*, 1988). Previous research on police employees has reported heavy alcohol consumption (Stevelink *et al.*, 2020; Syed *et al.*, 2020) and smoking (Stevelink *et al.*, 2020) to be associated with poorer mental health. Other research conducted on the general population has reported smoking to be associated with sickness absence (Laaksonen *et al.*, 2009; Troelstra *et al.*, 2020) as well as harmful drinking (Marzan *et al.*, 2022) and abstinence from alcohol (Marzan *et al.*, 2022). Health risk behaviours may be associated with sickness absence as they contribute to physical health problems, which leads to more sick days being taken. However, due to the cross-sectional nature of our data, this cannot be determined, and future research should investigate the relationship between health risk behaviours, mental health and physical health in police employees.

### Sickness absence and occupational stressors

Police employees with longer working hours had less sickness absence which may be related to occupational demands, as those with active job strain (high demand, high control) were also less likely to take sick leave. Previous research has reported that longer working hours increase the risk of psychological distress, emotional exhaustion and depersonalisation in police officers (Houdmont and Randall, 2016). However, as our data are cross-sectional it is difficult to determine the exact cause of the findings. Those affected by longer working hours may have already taken long-term sick leave and therefore, reported fewer working hours in the previous year. Police employees may have been working whilst having an illness, an issue of presenteeism, which has been reported for police officers in Sweden (Leineweber *et al.*, 2011) and Germany (Bachert *et al.*, 2017). In a UK study, 66% of police officers reported one or more episodes of presenteeism due to psychological health and the same percentage due to physical health within the previous year (Elliott-Davies, 2021a). It was not possible to explore presenteeism in our study.

Those with high and passive job strain had greater odds of taking sick leave. High job demand has been reported as a strong predictor of psychological distress in the general population (Nieuwenhuijsen *et al.*, 2010) and for police officers (Purba and Demou, 2019). Our results reflect those from research conducted on 290 Italian policemen that reported low control and high demand were associated with increased short-term sickness absence (Magnavita and Garbarino, 2013). However, this was a select group from a special police unit that had a very high amount of average sickness absence per policeman in 1 year (26 days) (Magnavita and Garbarino, 2013). Our findings highlight the need to address demand/control imbalances for police employees to prevent sickness absences related to job strain.

### Strengths and limitations

The main strength of our study is the use of a large and representative cohort of UK police employees to determine factors associated with sickness absence, an area that, to our knowledge, has previously been under-explored in this population. As the AHMS originally aimed to explore physical health effects from radio usage rather than occupational factors or sickness absence (Elliott *et al.*, 2014), participant responses may be less influenced by framing effects (Goodwin *et al.*, 2013).

The limitations of our study include not being able to determine the reasons why sickness absence was taken and using a self-reported measure for sickness absence which may have introduced bias. General population studies have reported differences between self-reported and employer-recorded sickness absence data, with self-reported sickness absence being under-reported (Ferrie *et al.*, 2005; Voss *et al.*, 2008).

Data were collected over a long period from 2006 to 2015 which may have introduced heterogeneity, for example in the outcome of interest. However, sickness absence rates have remained relatively stable over this time (Office for National Statistics, 2022). Due to the use of cross-sectional data and demographic differences in the sample (e.g., large participant numbers such as those from the Metropolitan Police Service only being included after 2011), it was not possible to determine the temporal associations between the specified variables with sickness absence (Elliott *et al.*, 2014). There may have been other reasons that led police employees to take sick leave which were not related to occupational factors (e.g., bereavement or physical health conditions).

A further limitation of our study is that sickness absence was retrospectively reported and the other explanatory variables were assessed at the time of data collection. Whilst the findings suggest associations between the explanatory variables and sickness absence, due to the variation of timings for data collection the findings do not allow for interpretations of the causality of the relationship. Further longitudinal research is required to determine the temporal relationship between them.

We did not include police employees’ job roles or their police force as these data were not provided. This information may have helped to determine whether certain police roles are more likely to take sick leave (e.g., traffic officers and crime scene investigators). There were many custom responses in the *all other ethnic groups combined* category which made it difficult to further define ethnicity. Lastly, it would be beneficial to consider how physical health conditions may impact sickness absence, which was beyond the scope of this study and warrants further exploration.

### Implications

Identifying the factors that contribute to sickness absence is vital for the development of interventions to support police employees to foster a healthier workforce and increase retention. The issue of presenteeism is one that particularly requires more focus as it is estimated that absences and presenteeism cost UK employers up to £45 billion annually due to employees’ working whilst suffering from poor mental health and as a result, have reduced productivity (Deloitte, 2020). Our findings indicated that police employees with poor mental health may be at a greater risk of taking sick leave. This population can be considered for targeted interventions, including all police employees and not just police officers, as police staff had more sickness absences in our study.

## Conclusions

Our study used cross-sectional data from the AHMS to explore factors associated with sickness absence among UK police employees. We found that over half of all police employees took at least 1 day of sickness absence in the previous year and a third between 1 and 5 days. Women, police staff and those with poor mental health (e.g., probable symptoms of depression, anxiety and PTSD) were more likely to take sick leave. Poorer job satisfaction and greater job strain were associated with more days of sickness absence. The findings highlight the importance of considering the factors that may contribute to sickness absence in police employees and is an area where more research is needed.

## Supporting information

Parkes et al. supplementary material Parkes et al. supplementary material

## Data Availability

The data that support the findings of this study are available through a formal application process (https://police-health.org.uk/applying-access-resource), but restrictions apply to the availability of these data, which were used under license for the current study, and so are not publicly available.

## Supplementary material

The supplementary material for this article can be found at https://doi.org/10.1017/S2045796024000283.

## References

[ref1] Allen G and Mansfield Z (2022) Police service strength. House of Commons Library. https://researchbriefings.files.parliament.uk/documents/SN00634/SN00634.pdf (accessed 21 December 2022).

[ref2] Bachert P, Walter UN and Mess F (2017) Presenteeism among German police officers: An empirical study on prevalence and reasons. *Prävention Und Gesundheitsf örderung* 12, 137–144.

[ref3] Benjamini Y and Hochberg Y (1995) Controlling the false discovery rate: A practical and powerful approach to multiple testing. *Journal of the Royal Statistical Society: Series B (Methodological)* 57, 289–300.

[ref4] Bjelland I, Dahl AA, Haug TT and Neckelmann D (2002) The validity of the Hospital Anxiety and Depression Scale: An updated literature review. *Journal of Psychosomatic Research* 52, 69–77. 11832252 10.1016/s0022-3999(01)00296-3

[ref5] Brewin CR (2005) Systematic review of screening instruments for adults at risk of PTSD. *Journal of Traumatic Stress* 18, 53–62. 16281196 10.1002/jts.20007

[ref6] Brewin CR, Rose S, Andrews B, Green J, Tata P, Mcevedy C, Turner S and Foa EB (2002) Brief screening instrument for post-traumatic stress disorder. *British Journal of Psychiatry* 181, 158–162. 10.1017/s0007125000161896 12151288

[ref7] Burchell B, Miller J, Brewin C, Soffia M and Wang S (2022) The association between job quality and the incidence of PTSD amongst police personnel. *Policing: A Journal of Policy and Practice* 17, paac054.

[ref8] Cartwright A and Roach J (2020) The wellbeing of UK police: A study of recorded absences from work of UK police employees due to psychological illness and stress using Freedom of Information Act data. *Policing: A Journal of Policy and Practice* 15, 1326–1338.

[ref9] Deloitte (2020) Mental health and employers – Refreshing the case for investment. https://www2.deloitte.com/content/dam/Deloitte/uk/Documents/consultancy/deloitte-uk-mental-health-and-employers.pdf (accessed 21 December 2022).

[ref10] Elliott-Davies M (2021a) Demand capacity & welfare survey 2020 headline report: PRRB. https://www.polfed.org/media/16557/dcw_prrb-report-13-01-2021-v20.pdf (accessed 21 December 2022).

[ref11] Elliott-Davies M (2021b) Demand capacity & welfare survey. Mental health and wellbeing support. https://www.polfed.org/media/17125/mental-health-and-wellbeing-support-june-2021-report.pdf (accessed 21 December 2022).

[ref12] Elliott P, Vergnaud A-C, Singh D, Neasham D, Spear J and Heard A (2014) The Airwave Health Monitoring Study of police officers and staff in Great Britain: Rationale, design and methods. *Environmental Research* 134, 280–285. 25194498 10.1016/j.envres.2014.07.025

[ref13] Fekedulegn D, Burchfiel CM, Hartley TA, Andrew ME, Charles LE, Tinney-Zara CA and Violanti JM (2013) Shiftwork and sickness absence among police officers: The BCOPS study. *Chronobiology International* 30, 930–941. 23808812 10.3109/07420528.2013.790043 PMC4624272

[ref14] Ferrie JE, Kivimäki M, Head J, Shipley MJ, Vahtera J and Marmot MG (2005) A comparison of self-reported sickness absence with absences recorded in employers’ registers: Evidence from the Whitehall II study. *Occupational & Environmental Medicine* 62, 74–79. 15657187 10.1136/oem.2004.013896 PMC1740949

[ref15] Golding SE, Horsfield C, Davies A, Egan B, Jones M, Raleigh M, Schofield P, Squires A, Start K, Quinn T and Cropley M (2017) Exploring the psychological health of emergency dispatch centre operatives: A systematic review and narrative synthesis. *PeerJ* 5, e3735. 10.7717/peerj.3735 PMC5649589 29062596

[ref16] Goodwin L, Ben-Zion I, Fear NT, Hotopf M, Stansfeld SA and Wessely S (2013) Are reports of psychological stress higher in occupational studies? A systematic review across occupational and population based studies. *PLoS One* 8, e78693. 10.1371/journal.pone.0078693 PMC3817075 24223840

[ref17] Her Majesty’s Inspectorate of Constabulary (HMIC) (1997) Lost time - The management of sickness absence and medical retirement in the police service.

[ref18] Home Office (2021) Police workforce, England and Wales: 31 March 2021 second edition. https://www.gov.uk/government/statistics/police-workforce-england-and-wales-31-march-2021/police-workforce-england-and-wales-31-march-2021 (accessed 13 June 2022).

[ref19] Houdmont J and Jachens L (2021) English police officers’ alcohol consumption and links with organisational job stressors. *The Police Journal* 95, 674–690.

[ref20] Houdmont J and Randall R (2016) Working hours and common mental disorders in English police officers. *Occupational Medicine* 66, 713–718. 27852878 10.1093/occmed/kqw166

[ref21] Hughes RA, Heron J, Sterne J and Tilling K (2019) Accounting for missing data in statistical analyses: Multiple imputation is not always the answer. *International Journal of Epidemiology* 48, 1294–1304. 30879056 10.1093/ije/dyz032 PMC6693809

[ref22] Irizar P, Gage SH, Fallon V and Goodwin L (2022) A latent class analysis of health risk behaviours in the UK Police Service and their associations with mental health and job strain. *BMC Psychiatry* 22, 426. 10.1186/s12888-022-04054-3 PMC9233366 35751116

[ref23] Irizar P, Gage SH, Field M, Fallon V and Goodwin L (2021a) The prevalence of hazardous and harmful drinking in the UK Police Service, and their co-occurrence with job strain and mental health problems. *Epidemiology and Psychiatric Sciences* 30, e51. 10.1017/S2045796021000366 PMC8220482 34402422

[ref24] Irizar P, Stevelink S, Pernet D, Gage SH, Greenberg N, Wessely S, Goodwin L and Fear NT (2021b) Probable post-traumatic stress disorder and harmful alcohol use among male members of the British Police Forces and the British Armed Forces: A comparative study. *European Journal of Psychotraumatology* 12, 1891734. 10.1080/20008198.2021.1891734 PMC8079084 33968324

[ref25] Karasek RA (1985). *Job content questionnaire and user’s guide*. Lowell: University of Massachusetts Lowell.

[ref26] Kessler RC, Berglund P, Demler O, Jin R, Merikangas KR and Walters EE (2005) Lifetime prevalence and age-of-onset distributions of DSM-IV disorders in the National Comorbidity Survey Replication. *Archives of General Psychiatry* 62, 593–602. 15939837 10.1001/archpsyc.62.6.593

[ref27] Körlin J, Alexanderson K and Svedberg P (2009) Sickness absence among women and men in the police: A systematic literature review. *Scandinavian Journal of Public Health* 37, 310–319. 19124595 10.1177/1403494808098508

[ref28] Kroenke K, Spitzer RL and Williams JBW (2001) The PHQ-9. *Journal of General Internal Medicine* 16, 606–613. 11556941 10.1046/j.1525-1497.2001.016009606.x PMC1495268

[ref29] Laaksonen M, Piha K, Martikainen P, Rahkonen O and Lahelma E (2009) Health-related behaviours and sickness absence from work. *Occupational & Environmental Medicine* 66, 840–847. 19934118 10.1136/oem.2008.039248

[ref30] Leineweber C, Westerlund H, Hagberg J, Svedberg P, Luokkala M and Alexanderson K (2011) Sickness presenteeism among Swedish police officers. *Journal of Occupational Rehabilitation* 21, 17–22. 20533079 10.1007/s10926-010-9249-1

[ref31] Magnavita N and Garbarino S (2013) Is absence related to work stress? A repeated cross-sectional study on a special police force. *American Journal of Industrial Medicine* 56, 765–775. 23334868 10.1002/ajim.22155

[ref32] Marzan M, Callinan S, Livingston M, Leggat G and Jiang H (2022) Systematic review and dose–response meta-analysis on the relationship between alcohol consumption and sickness absence. *Alcohol and Alcoholism* 57, 47–57. 33604615 10.1093/alcalc/agab008

[ref33] National Audit Office (2018) Financial sustainability of police forces in England and Wales 2018. https://www.nao.org.uk/wp-content/uploads/2018/09/Financial-sustainability-of-police-forces-in-England-and-Wales-2018.pdf (accessed 10 May 2023).

[ref34] Nieuwenhuijsen K, Bruinvels D and Frings-Dresen M (2010) Psychosocial work environment and stress-related disorders, a systematic review. *Occupational Medicine* 60, 277–286. 20511268 10.1093/occmed/kqq081

[ref35] Office for National Statistics (2022) Sickness absence in the UK labour market: 2021. https://www.ons.gov.uk/employmentandlabourmarket/peopleinwork/labourproductivity/articles/sicknessabsenceinthelabourmarket/2021/pdf (accessed 21 December 2022).

[ref36] Østby KA, Mykletun A and Nilsen W (2018) Explaining the gender gap in sickness absence. *Occupational Medicine* 68, 320–326. 29672758 10.1093/occmed/kqy062

[ref37] Pearce N, Checkoway H and Kriebel D (2007) Bias in occupational epidemiology studies. *Occupational & Environmental Medicine* 64, 562–568. 17053019 10.1136/oem.2006.026690 PMC2078501

[ref38] Pepinsky TB (2018) A note on listwise deletion versus multiple imputation. *Political Analysis* 26, 480–488.

[ref39] Purba A and Demou E (2019) The relationship between organisational stressors and mental wellbeing within police officers: A systematic review. *BMC Public Health* 19, 1286. 10.1186/s12889-019-7609-0 PMC6792329 31615479

[ref40] Shaper AG, Wannamethee G and Walker M (1988) Alcohol and mortality in British men: Explaining the U-shaped curve. *The Lancet* 332, 1267–1273. 10.1016/s0140-6736(88)92890-5 2904004

[ref41] Spitzer RL, Kroenke K, Williams JBW and Löwe B (2006) A brief measure for assessing Generalized Anxiety Disorder: The GAD-7. *Archives of Internal Medicine* 166, 1092–1097. 16717171 10.1001/archinte.166.10.1092

[ref42] Sterne J, White IR, Carlin JB, Spratt M, Royston P, Kenward MG, Wood AM and Carpenter JR (2009) Multiple imputation for missing data in epidemiological and clinical research: Potential and pitfalls. *BMJ* 338, b2393. 10.1136/bmj.b2393 PMC2714692 19564179

[ref43] Stevelink S, Opie E, Pernet D, Gao H, Elliott P, Wessely S, Fear NT, Hotopf M and Greenberg N (2020) Probable PTSD, depression and anxiety in 40,299 UK police officers and staff: Prevalence, risk factors and associations with blood pressure. *PLoS One* 15, e0240902. 10.1371/journal.pone.0240902 PMC7660485 33180769

[ref44] Svedberg P and Alexanderson K (2012) Associations between sickness absence and harassment, threats, violence, or discrimination: A cross-sectional study of the Swedish Police. *Work* 42, 83–92. 22635152 10.3233/WOR-2012-1333

[ref45] Syed S, Ashwick R, Schlosser M, Jones R, Rowe S and Billings J (2020) Global prevalence and risk factors for mental health problems in police personnel: A systematic review and meta-analysis. *Occupational & Environmental Medicine* 77, 737–747. 32439827 10.1136/oemed-2020-106498

[ref46] Troelstra SA, Coenen P, Boot CR, Harting J, Kunst AE and van der Beek AJ (2020) Smoking and sickness absence: Systematic review and meta-analysis. *Scandinavian Journal of Work, Environment & Health* 46, 5–18. 10.5271/sjweh.3848 31478055

[ref47] Violanti JM, Fekedulegn D, Hartley TA, Charles LE, Andrew ME, Ma CC and Burchfiel CM (2016) Highly rated and most frequent stressors among police officers: Gender differences. *American Journal of Criminal Justice* 41, 645–662. 28260848 10.1007/s12103-016-9342-x PMC5330309

[ref48] Voss M, Stark S, Alfredsson L, Vingård E and Josephson M (2008) Comparisons of self-reported and register data on sickness absence among public employees in Sweden. *Occupational & Environmental Medicine* 65, 61–67. 17704196 10.1136/oem.2006.031427

[ref49] Zigmond AS and Snaith RP (1983) The hospital anxiety and depression scale. *Acta Psychiatrica Scandinavica* 67, 361–370. 6880820 10.1111/j.1600-0447.1983.tb09716.x

