# Supplementary material for: Sickness absence and associations with sociodemographic factors, health risk behaviours, occupational stressors and adverse mental health in 40,343 UK police employees
Source: Epidemiol Psychiatr Sci. 2024 May 7;33:e26. doi: 10.1017/S2045796024000283 (PMC11094650; doi:10.1017/S2045796024000283)
Supplement: Parkes et al. supplementary material [file S2045796024000283sup001.docx]

**Sickness absence and associations with sociodemographic factors, health risk behaviours, occupational stressors, and adverse mental health in 40,343 UK police employees**

**Supplementary materials**

S. Parkes^1^

P. Irizar^2^

N. Greenberg^1^

S. Wessely^1^

N. T. Fear^1,4^

M. Hotopf^3,5^

S. A. M. Stevelink^1,3^

1. King’s Centre for Military Health Research, Institute of Psychiatry, Psychology & Neuroscience, King’s College London, London, United Kingdom.
2. Department of Sociology, School of Social Sciences, University of Manchester, Manchester, United Kingdom.
3. Department of Psychological Medicine, Institute of Psychiatry, Psychology & Neuroscience, King’s College London, London, United Kingdom.
4. Academic Department of Military Mental Health, Institute of Psychiatry, Psychology & Neuroscience, King’s College London, London, United Kingdom.
5. South London and Maudsley NHS Foundation Trust, London, United Kingdom.

Corresponding author: Steven Parkes, email: [steven.parkes@kcl.ac.uk](mailto:steven.parkes@kcl.ac.uk)

**Supplementary Table 1**. Multinomial logistic regression for health risk behaviours, occupational stressors, mental health outcomes, and sickness absence for police employees. Row frequencies and percentages are shown, along with multinomial odds ratios (MOR) and 95% confidence intervals (CI). Reference group for sickness absence is no sickness absence in the past year.

| **Characteristic** | **Days of sickness absence in the past year (*N* = 40,208)** | | | | | | |
| --- | --- | --- | --- | --- | --- | --- | --- |
|  | None  *n* (%) | Low (1-5)  *n* (%) | MOR  (95% CI) | Moderate (6-19)  *n* (%) | MOR  (95% CI) | Long-term sickness  absence (20 or more)  *n* (%) | MOR  (95% CI) |
| Overall (*n* = days of sick leave) | 18,572 (46.19) | 13,295 (33.07) |  | 5,189 (12.91) |  | 3,152 (7.84) |  |
| Rank |  |  |  |  |  |  |  |
| Police officer | 12,934 (49.77) | 7,979 (30.70) | 1.00 | 3,084 (11.87) | 1.00 | 1,989 (7.65) | 1.00 |
| Police staff | 4,045 (38.98) | 3,915 (37.72) | 1.57 (1.49, 1.65)*** | 1,579 (15.21) | 1.64 (1.53, 1.76)*** | 839 (8.08) | 1.35 (1.24, 1.47)*** |
| Gender |  |  |  |  |  |  |  |
| Male | 12,995 (51.31) | 7,775 (30.70) | 1.00 | 2,874 (11.35) | 1.00 | 1,684 (6.65) | 1.00 |
| Female | 5,576 (37.48) | 5,517 (37.09) | 1.65 (1.58, 1.73)*** | 2,315 (15.56) | 1.88 (1.76, 2.00)*** | 1,468 (9.87) | 2.03 (1.88, 2.19)*** |
| Age (in years) |  |  |  |  |  |  |  |
| < 30 | 2,071 (39.37) | 2,176 (41.36) | 1.00 | 731 (13.89) | 1.00 | 283 (5.38) | 1.00 |
| 30-39 | 5,534 (42.05) | 4,819 (36.62) | 0.83 (0.77, 0.89)*** | 1,820 (13.83) | 0.93 (0.84, 1.03) | 986 (7.49) | 1.30 (1.13, 1.50)*** |
| 40-49 | 8,036 (50.58) | 4,587 (28.87) | 0.54 (0.51, 0.58)*** | 1,911 (12.03) | 0.67 (0.61, 0.74)*** | 1,354 (8.52) | 1.23 (1.08, 1.41)** |
| 50+ | 2,931 (49.68) | 1,713 (29.03) | 0.56 (0.51, 0.61)*** | 727 (12.32) | 0.70 (0.63, 0.79)*** | 529 (8.97) | 1.32 (1.13, 1.54)*** |
| Ethnicity |  |  |  |  |  |  |  |
| White | 17,504 (46.18) | 12,570 (33.17) | 1.00 | 4,850 (12.80) | 1.00 | 2,977 (7.85) | 1.00 |
| All other ethnic groups combined | 967 (46.09) | 657 (31.32) | 0.95 (0.85, 1.05) | 313 (14.92) | 1.17 (1.02, 1.33)* | 161 (7.67) | 0.98 (0.82, 1.16) |
| Marital status |  |  |  |  |  |  |  |
| Married/Cohabiting | 14,697 (47.19) | 10,236 (32.86) | 1.00 | 3,859 (12.39) | 1.00 | 2,355 (7.56) | 1.00 |
| Divorced/Separated | 1,384 (42.72) | 1,033 (31.88) | 1.07 (0.98, 1.17) | 481 (14.85) | 1.32 (1.19, 1.48)*** | 342 (10.56) | 1.54 (1.36, 1.75)*** |
| Single | 2,033 (42.66) | 1,662 (34.87) | 1.17 (1.09, 1.26)*** | 700 (14.69) | 1.31 (1.19, 1.44)*** | 371 (7.78) | 1.14 (1.01, 1.28)* |
| Other | 403 (43.76) | 312 (33.88) | 1.11 (0.96, 1.29) | 134 (14.55) | 1.27 (1.04, 1.54)* | 72 (7.82) | 1.11 (0.86, 1.44) |
| Education |  |  |  |  |  |  |  |
| Low (O levels/GCSEs or none) | 6,055 (45.23) | 4,225 (31.56) | 1.00 | 1,841 (13.75) | 1.00 | 1,265 (9.45) | 1.00 |
| High (A levels, degree or higher) | 12,462 (46.70) | 9,018 (33.79) | 1.04 (0.99, 1.09) | 3,333 (12.49) | 0.88 (0.82, 0.94)*** | 1,875 (7.03) | 0.72 (0.67, 0.78)*** |
| Salary |  |  |  |  |  |  |  |
| < £25999 | 3,099 (36.48) | 3,381 (39.80) | 1.00 | 1,288 (15.16) | 1.00 | 727 (8.56) | 1.00 |
| £26000-£37999 | 6,953 (41.75) | 5,781 (34.71) | 0.76 (0.72, 0.81)*** | 2,460 (14.77) | 0.85 (0.79, 0.92)*** | 1,459 (8.76) | 0.89 (0.81, 0.99)* |
| £38000-£59999 | 7,525 (55.11) | 3,878 (28.40) | 0.47 (0.44, 0.50)*** | 1,355 (9.92) | 0.43 (0.40, 0.47)*** | 896 (6.56) | 0.51 (0.46, 0.56)*** |
| > £60000 | 940 (73.90) | 203 (15.96) | 0.20 (0.17, 0.23)*** | 71 (5.58) | 0.18 (0.14, 0.23)*** | 58 (4.56) | 0.26 (0.20, 0.35)*** |
| Total hours worked per week (inc. overtime) |  |  |  |  |  |  |  |
| ≤ 40 hours | 6,798 (41.09) | 5,756 (34.79) | 1.00 | 2,348 (14.19) | 1.00 | 1,641 (9.92) | 1.00 |
| 41-48 hours | 6,278 (47.03) | 4,491 (33.65) | 0.84 (0.80, 0.89)*** | 1,690 (12.66) | 0.78 (0.73, 0.84)*** | 889 (6.66) | 0.59 (0.54, 0.64)*** |
| ≥ 49 hours | 5,216 (53.65) | 2,864 (29.46) | 0.65 (0.61, 0.69)*** | 1,071 (11.02) | 0.59 (0.55, 0.64)*** | 572 (5.88) | 0.45 (0.41, 0.50)*** |
| Years in police force |  |  |  |  |  |  |  |
| ≤ 5 years | 3,266 (39.70) | 3,324 (40.40) | 1.00 | 1,131 (13.75) | 1.00 | 506 (6.15) | 1.00 |
| 6-10 years | 3,630 (39.96) | 3,328 (36.63) | 0.90 (0.84, 0.96)** | 1,363 (15.00) | 1.08 (0.99, 1.19) | 764 (8.41) | 1.36 (1.20, 1.53)*** |
| 11-20 years | 5,813 (46.59) | 4,005 (32.10) | 0.68 (0.64, 0.72)*** | 1,611 (12.91) | 0.80 (0.73, 0.87)*** | 1,049 (8.41) | 1.16 (1.04, 1.31)** |
| > 20 years | 5,840 (56.35) | 2,622 (25.30) | 0.44 (0.41, 0.47)*** | 1,076 (10.38) | 0.53 (0.48, 0.58)*** | 826 (7.97) | 0.91 (0.81, 1.03) |
| Smoking status |  |  |  |  |  |  |  |
| Non-smoker | 17,177 (47.02) | 11,983 (32.80) | 1.00 | 4,572 (12.52) | 1.00 | 2,800 (7.66) | 1.00 |
| Current smoker | 1,330 (37.97) | 1,241 (35.43) | 1.34 (1.23, 1.45)*** | 591 (16.87) | 1.67 (1.51, 1.85)*** | 341 (9.73) | 1.57 (1.39, 1.78)*** |
| Alcohol consumption |  |  |  |  |  |  |  |
| Non-drinker | 1,551 (42.41) | 1,161 (31.75) | 0.99 (0.91, 1.08) | 540 (14.77) | 1.22 (1.10, 1.36)*** | 405 (11.07) | 1.59 (1.41, 1.79)*** |
| Low risk | 10,022 (45.36) | 7,569 (34.26) | 1.00 | 2,855 (12.92) | 1.00 | 1,647 (7.45) | 1.00 |
| Hazardous | 6,379 (48.90) | 4,104 (31.46) | 0.85 (0.81, 0.89)*** | 1,615 (12.38) | 0.89 (0.83, 0.95)** | 947 (7.26) | 0.90 (0.83, 0.98)* |
| Harmful | 542 (44.83) | 380 (31.43) | 0.93 (0.81, 1.06) | 146 (12.08) | 0.95 (0.78, 1.14) | 141 (11.66) | 1.58 (1.31, 1.92)*** |
| Binge drinking |  |  |  |  |  |  |  |
| Binge drinks less than 2-4 times a month | 12,767 (45.76) | 9,314 (33.39) | 1.00 | 3,625 (12.99) | 1.00 | 2,192 (7.86) | 1.00 |
| Binge drinks at least 2-4 times a month | 5,727 (47.30) | 3,901 (32.22) | 0.93 (0.89, 0.98)** | 1,531 (12.65) | 0.94 (0.88, 1.01) | 948 (7.83) | 0.96 (0.89, 1.05) |
| GP consultations (in the past year) |  |  |  |  |  |  |  |
| 0 | 7,490 (19.95) | 3,392 (9.03) | 0.52 (0.49, 0.55)*** | 462 (4.04) | 0.18 (0.17, 0.20)*** | 92 (0.25) | 0.10 (0.08, 0.12)*** |
| 1-2 | 7,798 (20.77) | 6,786 (18.07) | 1.00 | 2,623 (14.40) | 1.00 | 1,002 (2.67) | 1.00 |
| 3-4 | 1,487 (3.96) | 1,702 (4.53) | 1.32 (1.22, 1.42)*** | 1,214 (23.07) | 2.43 (2.22, 2.65)*** | 860 (2.29) | 4.50 (4.05, 5.01)*** |
| 5+ | 467 (1.24) | 588 (1.57) | 1.45 (1.28, 1.64)*** | 566 (21.47) | 3.60 (3.16, 4.10)*** | 1,015 (2.70) | 16.91 (14.89, 19.22)*** |
| Mental health |  |  |  |  |  |  |  |
| PTSD case | 560 (37.91) | 427 (28.91) | 1.06 (0.93, 1.21) | 259 (17.54) | 1.69 (1.45, 1.97)*** | 231 (15.64) | 2.58 (2.20, 3.02)*** |
| Depression case | 1,291 (33.32) | 1,204 (31.07) | 1.33 (1.23, 1.45)*** | 734 (18.94) | 2.21 (2.01, 2.43)*** | 646 (16.67) | 3.46 (3.12, 3.84)*** |
| Anxiety case | 1,190 (35.51) | 1,060 (31.63) | 1.26 (1.16, 1.38)*** | 587 (17.52) | 1.87 (1.68, 2.07)*** | 514 (15.34) | 2.85 (2.55, 3.19)*** |
| Job satisfaction |  |  |  |  |  |  |  |
| Very satisfied | 4,543 (55.72) | 2,377 (29.15) | 0.71 (0.67, 0.75)*** | 777 (9.53) | 0.61 (0.56, 0.67)*** | 456 (5.59) | 0.62 (0.56, 0.70)*** |
| Satisfied | 11,111 (45.89) | 8,212 (33.91) | 1.00 | 3,103 (12.81) | 1.00 | 1,788 (7.38) | 1.00 |
| Dissatisfied | 2,204 (37.22) | 2,105 (35.55) | 1.29 (1.21, 1.38)*** | 963 (16.26) | 1.56 (1.44, 1.70)*** | 650 (10.98) | 1.83 (1.66, 2.03)*** |
| Very dissatisfied | 434 (32.75) | 417 (31.47) | 1.30 (1.13, 1.49)*** | 266 (20.08) | 2.19 (1.87, 2.57)*** | 208 (15.70) | 2.98 (2.51, 3.54)*** |
| Job strain |  |  |  |  |  |  |  |
| Low | 5,348 (49.69) | 3,502 (32.54) | 1.00 | 1,226 (11.39) | 1.00 | 686 (6.37) | 1.00 |
| High | 3,867 (40.30) | 3,396 (35.39) | 1.34 (1.26, 1.43)*** | 1,436 (14.97) | 1.62 (1.49, 1.77)*** | 896 (9.34) | 1.81 (1.62, 2.01)*** |
| Active | 5,787 (52.39) | 3,309 (29.96) | 0.87 (0.82, 0.93)*** | 1,222 (11.06) | 0.92 (0.84, 1.01) | 728 (6.59) | 0.98 (0.88, 1.10) |
| Passive | 3,290 (40.07) | 2,904 (35.37) | 1.35 (1.26, 1.44)*** | 1,225 (14.92) | 1.62 (1.48, 1.78)*** | 792 (9.65) | 1.88 (1.68, 2.10)*** |

**p* < 0.05, ***p* < 0.01, ****p* < 0.001.
